# Professor Caldwell and Animal Chemistry

**Published:** 1844-06

**Authors:** 


### PROFESSOR CALDWELL AND ANIMAL CHEMISTRY.

Some of our friends, very recently, have expressed to us an apprehension that the spirit of our review of Professor Caldwell's Critique on Liebig's Animal Chemistry, might be misconceived. To prevent the possibility of any such misconception, we gladly avail ourselves of this opportunity to declare, that our review was written in a temper of the utmost respect and kindness for our distinguished colleague, whose magnanimity, we knew, tolerated the severest criticism of his doctrines. If our notice contains a single expression which evinces anything of a contrary spirit, we sincerely regret it. We would gladly expunge every word, which might not be used with propriety by a pupil towards a venerated preceptor. We felt constrained to notice the Critique, and, in doing so, we set forth plainly our objections to it; at the same time, it was our study to avoid every form of expression that could give offence. Y.

